# Research on wind comfort regulation strategies for public spaces in aged residential communities of cold regions during windy spring seasons

**DOI:** 10.1371/journal.pone.0331653

**Published:** 2025-10-31

**Authors:** Wen Xue, Xiaojun Guo, Xiaodan Chen, Yuanfeng Wang, Rui Chai, Yichao Wang, Yu Xia, Qianjun Cai

**Affiliations:** 1 School of Architecture and Art, Hebei University of Architecture, Zhangjiakou, Hebei, China; 2 Hebei Collaborative Innovation Center of Green Buildings, Zhangjiakou, China; Universidad Tecnica Federico Santa Maria, CHILE

## Abstract

In this study, the outdoor space of an aged residential community in Zhangjiakou, China, where strong winds frequently occur during the spring season, was investigated for environmental modifications. The study employed Rayman software to analyze the acceptable outdoor wind comfort range for residents, and utilized EDDY 3D simulation software to simulate and assess the current outdoor wind environment in the district. The analysis revealed that wind comfort was inadequate in several outdoor activity areas. The study suggests that the combination of landscape walls, enclosed spaces, and windbreak plants can effectively enhance wind environment conditions. The results indicate that: (1) The opening at the bottom of the wind-blocking wall can improve the static wind area at the corner, promote air circulation, and prevent pollutant accumulation. The larger the opening, the broader the influence of the wind shadow area and the greater the wind comfort area. (2) In cubic outdoor enclosed spaces, the degree of enclosure affects wind field conditions. Among these, the primary factor in enhancing the wind comfort area is the level and quantity of shelters perpendicular to the wind direction; the wind velocity variation in the wind shadow area is positively correlated with the degree of space enclosure. (3) Among the combinations of windbreak plants, the landscape configurations featuring equal height and gradient elevation arrangements. (4) After implementing the above three strategies to renovate the outdoor space of aged residential community, a computer simulation indicated that, under prevailing spring wind conditions, the wind-comfortable outdoor area increased from 40.26% to 79.84% demonstrate superior wind protection efficacy.

## 1. Introduction

In recent years, the rapid development of cities and human activities have significantly impacted the climate, with the frequent occurrence of extreme weather severely affecting residents’ quality of life [1]. Additionally, the ‘Venturi effect’ has emerged as one of the new forms of urban climate disasters [2]. Particularly in high-density residential areas, where strong winds are common, the row layout of older residential communities causes an increase in wind velocity in outdoor public spaces due to the building form [3]. This can negatively impact the comfort of residents during outdoor activities and, in extreme cases, lead to the disintegration or collapse of building structures. Therefore, addressing the issues related to urban climate environments is crucial [4,5].

Research on the outdoor wind environment primarily focuses on two aspects: the comfort evaluation system and the enhancement of design methods.

The outdoor wind environment evaluation system was initially based on the Beaufort wind scale, proposed by Francis Beaufort, a British scientist, in 1806 [6]. It is divided into 13 levels to assess the behavioral responses of individuals to different wind velocities. Subsequent studies have further refined the wind comfort range based on the Beaufort wind scale, considering wind velocities below 5 m/s as the acceptable range for wind comfort [7–11]. This range corresponds to the wind velocity defined based on the physiological responses to mechanical forces exerted by wind on the human body, whereas human comfort in outdoor environments is primarily represented by thermal comfort indices. The concept of equivalent temperature (ET) was first proposed by ASHRAE in 1923 [12], and since then, approximately 165 thermal indices have been developed to assess human comfort [13]. Current indices commonly used for outdoor thermal comfort include PET (including mPET), UTCI, PMV, SET, and THI, with PET and UTCI being the most widely applied. The factors influencing thermal comfort are complex, encompassing regional climatic differences, solar radiation, seasonal variations in wind velocity, clothing, personal metabolic rate, and subjective thermal sensations [14]. Additionally, when determining acceptable wind velocity ranges in outdoor environments, it is crucial to consider various environmental attributes.

Research on design methodologies for improving the outdoor wind environment in settlements has primarily focused on building morphology and layout. Tsichritzis’s research indicates that increasing building height leads to higher upper-level winds at pedestrian level, thereby expanding the range of high wind velocities at those heights [15]. Du et al. found that the porosity between buildings influences wind velocity, with larger porosities resulting in lower wind velocities compared to smaller ones [16]. Tamura’s findings indicated that increasing a building’s width enhances its wind-blocking effect, thereby creating a larger area of reduced wind velocity [17]. Juan et al. found that increasing the length and radius of a building’s chamfer at its edges reduced the intensity and extent of high-velocity turbulence, with rounded corners yielding the greatest effect [18]. According to Lin et al., when a building’s long side is oriented parallel to the wind direction, wind velocity around the building increases. Conversely, when oriented perpendicular to the wind, surrounding wind velocity decreases [19]. Tetsu et al. conducted an experiment that found a significant negative correlation between building density and volume and the wind environment in residential areas. This suggests that as building density and volume increase within a given area, the average wind velocity decreases [20]. Mixed and closed settlement layouts were found to obstruct incoming external winds, thereby creating a more favorable internal wind environment compared to a row layout [21].

All the above methods are used to improve the wind comfort of the surrounding space by changing the properties of the building itself. However, due to policy and economic considerations, aged residential community projects frequently utilize small-scale micro-renewal designs [22–24]. Yuan’s proposition asserts that the incorporation of vegetation can augment ground roughness, curtail outdoor wind velocity, and enhance the microclimate of urban spaces [25]. Hu’s research indicates that outdoor vegetation also affects the wind environment, with spaces covered by evergreen plants exhibiting lower wind velocities compared to deciduous plant spaces [26]. Parinaz’s research further explores the effect of pavement characteristics on wind behavior, proposing that the height, rotation angle, and spacing of pavement walls influence the average wind velocity in street spaces [27]. Liu et al. proposed a multifaceted approach, integrating low-albedo flooring materials, green configurations, and amenity features, such as wind walls, to enhance outdoor comfort [28].

Existing studies have quantitatively examined microclimate environments, outdoor environments, and human physiological comfort. However, these studies have not sufficiently considered specific design solutions. Additionally, the study’s geographical location is noteworthy, as it is situated in a cold region characterized by year-round windiness, particularly during the spring. This region has received limited attention in academic literature, particularly in relation to specific optimization solutions. Therefore, there is a clear need for further research on specific solutions for microclimate optimization in outdoor spaces within this climate type. The potential benefits of such research are numerous, including enhanced outdoor comfort within settlements, increased resident participation in outdoor activities, and improved health outcomes. Urban and architectural designers can also use the research findings to optimize and update design solutions, creating greener and more livable community environments.

The core of outdoor wind environment regulation lies in reducing near-ground wind velocity through momentum transfer and energy dissipation. Windbreak walls, architectural spaces, and landscape vegetation are fundamental components of urban open spaces and all contribute to wind mitigation by fulfilling the above physical mechanisms. As solid barriers, windbreak walls create high-pressure zones on the windward side and low-pressure zones on the leeward side. Airflow is redirected as it passes through the wall, generating a wake vortex region behind it, where turbulence mixing further dissipates energy. The porosity of the wall is a key parameter in controlling both the extent and rate of wind velocity attenuation [29]. Semi-enclosed spaces guide airflow separation through geometric configurations: sharp edges induce large-scale vortices, while internal cavities promote secondary circulation. This process continuously dissipates kinetic energy through shear stress while suppressing local acceleration effects [30,31]. Vegetation systems, as biologically porous media, generate distributed surface resistance through their canopy branches and leaves, directly absorbing wind momentum. They also increase surface roughness, alter boundary layer velocity profiles, and enhance energy dissipation through leaf vibration and small-scale turbulence [32].

This study adopts an integrated strategy of wall, enclosure, and vegetation elements, based on the following three core considerations:

1. In conventional renovation strategies, fully enclosed structures may block wind completely but severely compromise the openness and natural ventilation of outdoor spaces. Topographic modifications require extensive earthworks and disrupt existing infrastructure. A dense vegetation-only approach requires a long maturation period and may obstruct the understory space. In contrast, the integrated strategy enhances effectiveness through multi-element synergy: walls offer immediate wind protection, enclosure structures optimize the local airflow field, and vegetation contributes to multi-layered wind energy dissipation. Together, these elements produce complementary effects across spatial and temporal dimensions.
2. In high-density built environments, this design employs parametric control to balance protective performance and spatial occupation. For example, wall porosity is adjusted to regulate wind velocity attenuation gradients, the opening ratio of enclosures controls internal ventilation efficiency, and vegetation density modulates the near-ground wind environment. This flexible design can be adapted to constrained sites and irregular urban plots, avoiding the irreversibility of topographic modifications and the spatial deprivation caused by full enclosures.
3. Vegetation systems serve not only as wind-control components but also provide ecosystem services such as carbon sequestration, oxygen release, urban heat island mitigation, and biodiversity enhancement [33]. This dual-function integration of wind control and ecological benefits aligns the strategy with nature-based solution principles and maximizes environmental benefits over the project’s life cycle.

## 2. Methods

### 2.1. Purpose and procedure

**Fig 1:**
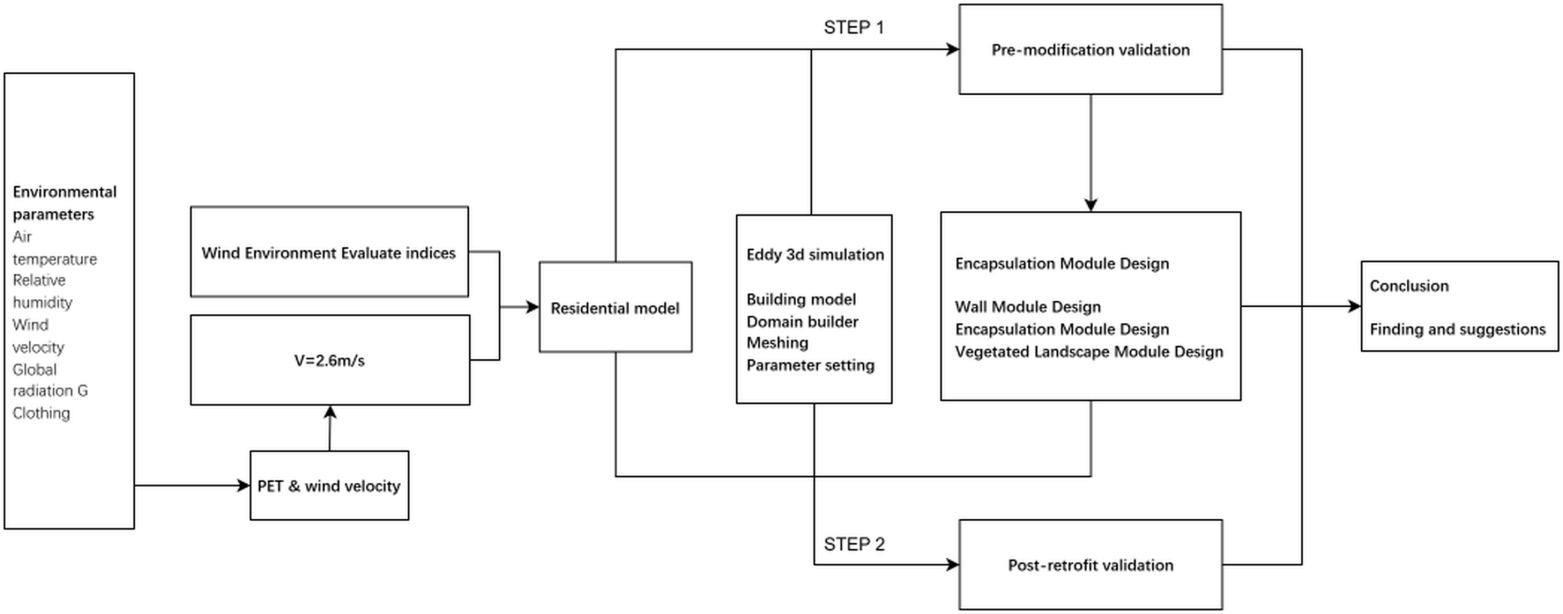
Research framework.

This study focuses on an aged residential community in Zhangjiakou City, a region characterized by severe cold weather conditions in China. The residents of this community, observed in their everyday attire, serve as the subjects of this study. The integration of field research methodologies with computer-based CFD simulations and analysis techniques enables the examination of the relationship between outdoor wind velocity and PET, as well as the distribution of the wind field within the actual environment. The subsequent analysis results in the formulation of strategic measures for the region, aimed at enhancing wind comfort in outdoor environments. The specific research steps are illustrated in Fig 1.

1. The Rayman software was used to analyze the acceptable wind comfort range of residents during strong spring winds in conjunction with daily attire and behavioural activities [34].
2. A detailed measurement of the site and buildings was conducted within the boundaries of the Explorer Dormitory.A 3D model was subsequently established, and a thorough analysis of the current situation was conducted using the relevant CFD simulation and modelling software [35].
3. In regions where wind comfort falls short of the requisite standard, an optimization design strategy is formulated. This strategy involves simulating 12 micro-modification scenarios for the outdoor wind environment across three levels. The optimization effects of the scenarios are evaluated using one thermal environment index (PET) and four wind environment evaluation metrics: Comfort Wind Area, Average Wind Velocity, Mean Velocity Ratio, and Wind Velocity Standard Deviation.
4. The site was renovated and updated in order to verify the feasibility of the program, based on relevant outdoor environmental evaluation indicators [36].

### 2.2. Background overview and in-situ experimental study

#### 2.2.1. Background of the study.

**Fig 2:**
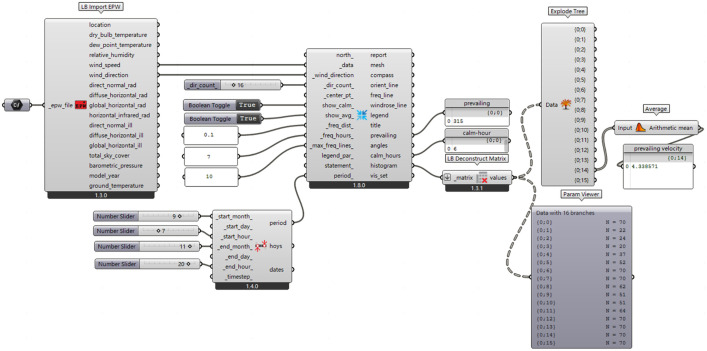
Annual wind direction and velocity analysis program.

The aged residential community under study is located in Zhangjiakou City (40°47′N, 114°53′E), in northern China. Geographically, Zhangjiakou is situated near the Inner Mongolia Plateau, which is influenced by the Siberian monsoon. The large expanse of grassland does not significantly weaken the wind, resulting in stronger winds. Regarding the terrain, Zhangjiakou is located at the junction of the Yanshan and Taihang Mountains, forming a long, narrow strip of land. The Venturi effect amplifies the wind velocity in this region. As shown in Fig 2, EPW meteorological data from 2007 to 2021 were analyzed using Ladybug tools, and the results are presented in Table 1. The dominant wind direction throughout the year is northwest (315°), and wind resources are abundant year-round [38]. In terms of seasonal distribution, due to the spring temperature rebound, varying altitudes in the mountainous terrain are heated unevenly by solar radiation, leading to changes in the surface air pressure gradient and the formation of strong winds. Consequently, spring wind potential, influenced by the monsoon, is greater, with daytime wind velocities in May reaching up to 6.03 m/s. This wind environment affects the comfort of residents during outdoor activities [39]. Therefore, May is considered a typical month for this study.

**Table 1:** Results of wind direction and wind velocity analysis within the baseThis study focuses on the Tanji Dormitory District at the junction of Industrial East Street and Shengli North Road in Qiaodong District, Zhangjiakou City, as shown in Fig 3. The area contains eight existing six-storey residential buildings, each with a height of 24 m, arranged in a row-type layout. A narrow tube-like space is formed between the buildings, and when the dominant monsoon wind flows through this gap, the local wind velocity increases dramatically due to the Venturi Effect [37]. Prolonged exposure to strong winds can cause discomfort such as breathlessness, headaches, and joint pain, which greatly inconveniences residents’ movement and outdoor activities.

Month	1	2	3	4	5	6	7	8	9	10	11	12
**Wind velocity (m/s)**	3.45	4.56	4.46	5.52	6.03	3.65	3.69	4.06	2.46	3.99	4.33	3.93
**Wind direction**	337.5	315	315	315	315	157.5	135	135	337.5	315	315	315

**Fig 3:**
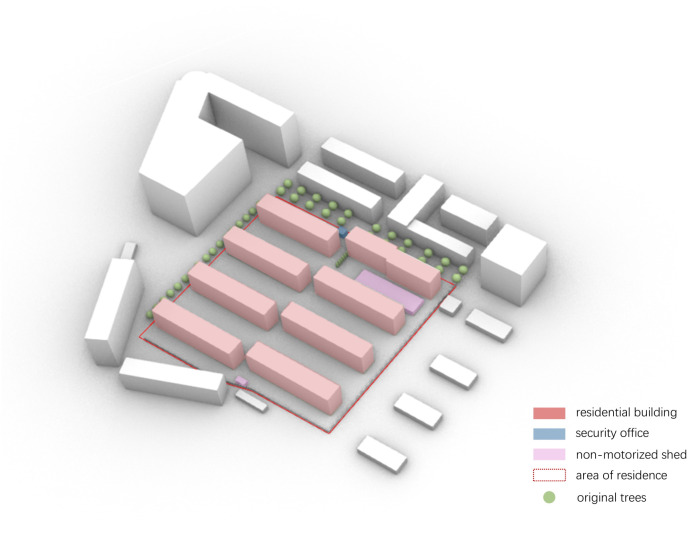
Study of the current state of the buildings and environment within the site.

#### 2.2.2 In-situ experimental study.

This site was selected as the experimental field to assess the effectiveness of wind environment regulation strategies under typical residential spatial conditions.

The ground-level space between buildings is primarily used for resident recreation and temporary parking, supplemented by several deciduous trees. Due to the transient nature of parked vehicles and the limited size and density of existing vegetation, their impact on airflow obstruction is minimal and can be considered negligible.

**Fig 4:**
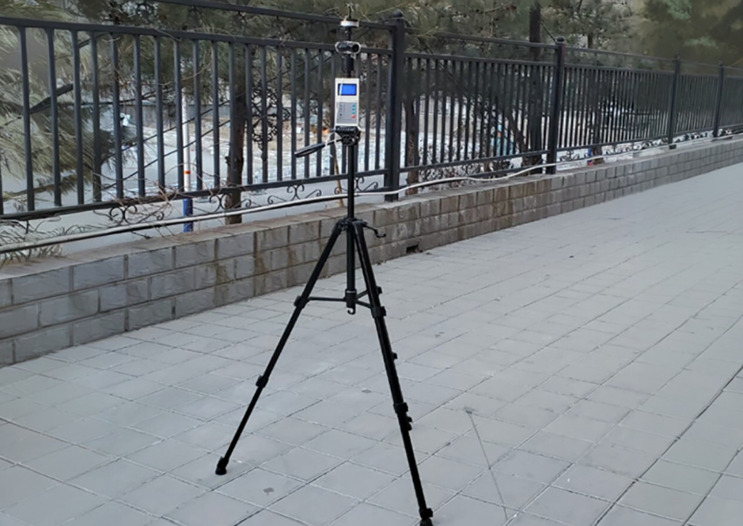
Portable weather station.

**Fig 5:**
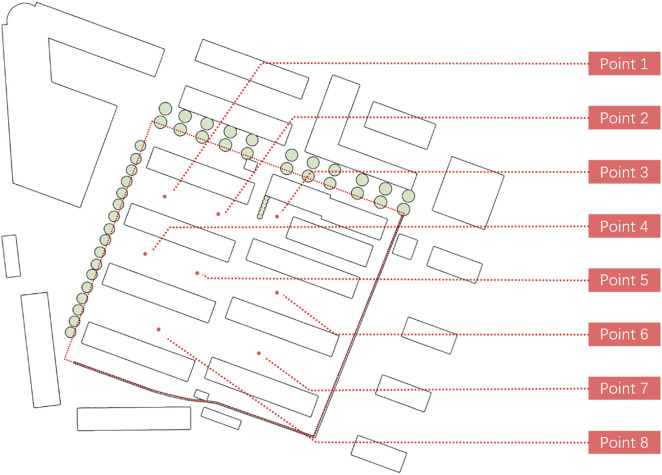
Measurement points in the TanJi Dormitory Community.

As shown in the Fig 4, this study employed a portable weather station to measure relevant parameters, including wind velocity (range: 0–30 m/s; accuracy: ± 0.3 m/s; resolution: 0.01 m/s) and wind direction (16 directions; accuracy: ± 1°; resolution: 1°). Fig 5 shows 8 measurement points were installed, prioritizing locations where residents typically engage in outdoor activities, to cover the core areas of communal use within the community, in order to investigate the impact of airflow on residents’ daily activities. The measured data were quantitatively compared with Eddy3D simulation results obtained under identical spatial positions and meteorological conditions. By comparing measured and simulated wind velocity and direction, the accuracy of the CFD model in simulating the wind environment of the site was evaluated, thereby validating its effectiveness and reliability for assessing wind conditions in residential activity areas.

### 2.3. Simulation software selection

**Fig 6:**
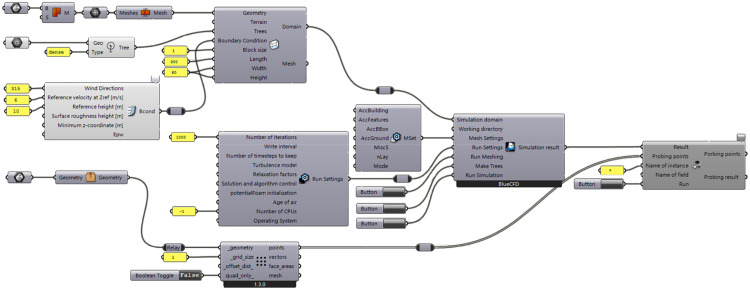
Procedures for regional wind environment analysis.

Computational fluid dynamics (CFD) is widely used in engineering applications within building design to simulate both indoor and outdoor natural ventilation due to its high accuracy and ease of use [40]. Numerous CFD-related simulation software are available. Among them, Eddy 3D [41] and Ladybug Tools [42] are CFD simulation plug-ins based on the OpenFOAM solver. These tools can simulate the wind environment of both building structures and plant landscapes. They operate within the widely used parametric design platforms Rhino and Grasshopper. These tools are capable of visualizing the calculation results effectively, and a large body of literature and experiments has verified the accuracy and reliability of the results [43]. Therefore, Eddy 3D and Ladybug Tools are used as the solver and visualization tools, respectively, for this study, with the calculation steps shown in Fig 6.

### 2.4. Setting of the calculation conditions

#### 2.4.1. Setting of the basic calculation conditions.

**Fig 7:**
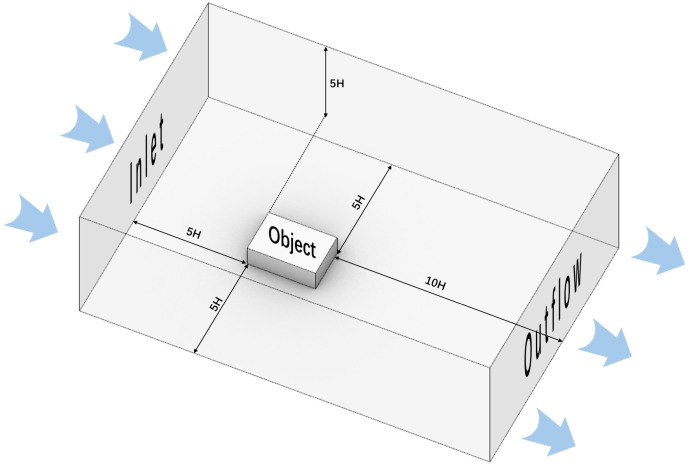
Calculation of the simulation area.

EDDY 3D software models the physical field using the RANS equations in CFD simulations, with the RNG k-ε turbulence model selected for numerical calculations. The boundary conditions play a crucial role in determining the accuracy of the results [44]. According to the specification for meteorological parameters of wind environments around buildings (JGJ/T449-2018), the 3D model scale was set to 1H-2H around the study area. The model includes the main buildings and trees with a continuous planting height of at least 3 m. The highest point of the 3D model was used as the minimum calculation boundary. The entrance boundary distance is 5H, the exit boundary distance is 10H, and the height boundary distance is 5H, as shown in Fig 7.

The number of computational meshes significantly influences the accuracy of the results [45]. In this simulation, the box-shaped domain module in EDDY 3D automatically generates the computational mesh, which is encoded at three levels in the mesh setting module [46]. The minimum cell size is adjusted according to the dimensions of the simulation scheme. And the minimum grid resolution of the actual building complex should be set to about 0.1 times of the buildings in the area including the evaluation points around the target buildings (about 0.5–5.0 m) [47]. The simulation begins with 2000 iterations, and the calculation stops once the results converge.

#### 2.4.2. Grid convergence and independence validation.

**Fig 8:**
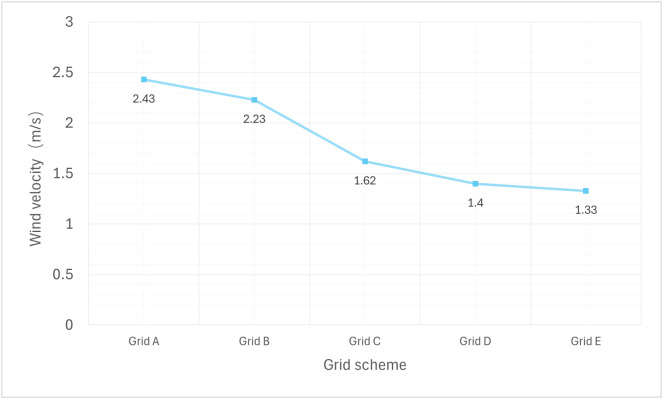
Grid convergence plot.

Before the formal grid uncertainty analysis, we first conducted a preliminary grid sensitivity study. The aim was to assess, in a straightforward manner, how simulation results depend on grid density, and to identify a suitable grid for the GCI analysis. As shown in Table 2, five grids with different densities were generated, ranging from the coarsest (Grid A) to the finest (Grid E). The wind velocity at a height of 1.5 m at point 1, obtained from field measurements, was chosen as the monitoring parameter. Simulations were performed for all five grids. As illustrated in Fig 8, the monitored wind velocity gradually stabilized as the number of grid nodes increased. When the total cell count exceeded 2.09 × 10⁶, the variation in wind velocity decreased significantly, indicating that the solution had begun to converge.

**Table 2:** Details of grid division for simulation schemes.

Grid scheme	Grid A	Grid B	Grid C	Grid D	Grid E
**Number of cells**	0.62 × 10^6^	0.93 × 10^6^	1.39 × 10^6^	2.09 × 10^6^	3.14 × 10^6^

The results of computational fluid dynamics simulations must be verified to ensure they are not influenced by grid resolution. In this study, we adopted the Grid Convergence Index (GCI) method, as recommended by the ASME V&V 20 Committee and widely recognized in the field. This method requires simulations to be performed on at least three systematically refined grids. Based on the observations from Fig 8, we selected the three grids exhibiting the most stable convergence behavior (i.e., Grid C, Grid D, and Grid E in the figure) for the subsequent GCI analysis. These were designated as the coarse grid (Grid 3), medium grid (Grid 2), and fine grid (Grid 1), respectively. The corresponding representative grid sizes were denoted as h1, h2, and h3. The formulas used for the GCI calculations are presented in Table 3.

**Table 3:** Grid convergence evaluation indicators.

Environmental Indicator	Formula	Theoretical significance	Evaluation criteria
Grid refinement ratio	$r=h_{\text{2}}/h_{\text{1}}=h_{\text{3}}/h_{\text{2}}$	Ensures the grid refinement is significant enough to capture the systematic error variation.	The value should be greater than 1.3 to ensure the analysis is reliable.
Apparent order of convergence	$p=\frac{\text{1}}{\text{ln}(r)}\left|\text{ln}\left|\frac{\varphi_{\text{3}}-\varphi_{\text{2}}}{\varphi_{\text{2}}-\varphi_{\text{1}}}\right|\right|$	Verifies a stable and predictable convergence rate, which validates the error analysis	A value near the theoretical order of 2 indicates good convergence.
Grid Convergence Index	$GCI_{fine}=\frac{F_{s}}{r^{p}-\text{1}}\times \left|\frac{\varphi_{\text{1}}-\varphi_{\text{2}}}{\varphi_{\text{1}}}\right|$	Provides a conservative estimate of the discretization error in the fine-grid solution.	A small GCI value (typically < 5%) indicates a small discretization error and high confidence in the reliability of the numerical results.

Note:

ϕ1, ϕ2, and ϕ3: The solutions for a monitored variable obtained on the fine, medium, and coarse grids, respectively.

Fs: The Factor of Safety is taken as 1.25, a recommended value for studies involving three or more grids.

### 2.5. Wind environment evaluation criteria

Environmental comfort at pedestrian height is influenced by both wind and thermal environments [48,49]. However, Liu, Jin et al. concluded that in cold regions, the primary factor affecting outdoor human comfort is solar radiation, while the secondary factor is the prevailing monsoon wind velocity, which remains the dominant factor under consistent solar radiation [27,50].

This paper selects one thermal environment indicator, physiological equivalent temperature (PET), and four wind environment indicators: area of wind comfort, average wind velocity, wind velocity ratio, and wind velocity dispersion, to evaluate outdoor comfort [51], as shown in the Table 4.

**Table 4:** Wind environment evaluation indicators.

Environmental Indicator	Formula	Theoretical significance	Evaluation criteria
PET(°C)	PET=Ta+(Hc−He)×K	Human physiological responses under conditions of integrated human activity and environmental parameters	PET between 13–29°C is considered to be an acceptable interval [52]
Area of wind comfort(㎡)	$S_{c}=S_{p\text{2}.\text{6}}\times S^{\text{2}}$	Cumulative area of wind comfort zone (v < 2.6m/s)	The larger the area, the more favourable it is for residents’ outdoor behaviour.
Average wind velocity(m/s)	$u=\frac{\text{1}}{n}\sum_{i=\text{1}}^{n}\;x_{i}$	Average of pedestrian-level wind velocities at a height of 1.5 m	The smaller the value, the lower the overall wind velocity in the wind park.
Average wind velocity ratio	$VR_{W}=\frac{V_{i}}{V_{\text{0}}}$	Average rate of change of pedestrian-level wind velocity at a height of 1.5 m	The smaller the value, the more effective the facility is at reducing wind velocity.
Wind-velocity dispersion	$\beta =\sqrt{\frac{\text{1}}{n}\sum_{i=\text{1}}^{n}\;(x_{i}-\mu {)}^{\text{2}}}$	Degree of variability in wind velocity	A smaller standard deviation indicates a more balanced wind velocity in the wind field.

Note:

Ta: Air temperature.

$H_{c}$: Metabolic heat production rate of the human body.

He: the rate of heat dissipation by the body through radiative and convective heat transfer.

K: a constant,indicating the thermal sensitivity of the body.

$S_{p\text{2}.\text{6}}$: the area of pixels in a point surface with a point value less than 2.6.

$S^{\text{2}}$: a scale factor,depends on the ratio of the resolution of the point surface to the actual measurement area.

$V_{i}$: Wind velocity at 1.5 m pedestrian level at a point in the actual spatial site.

$V_{\text{0}}$: the unaffected wind velocity at the same spatial coordinates.

**Fig 9:**
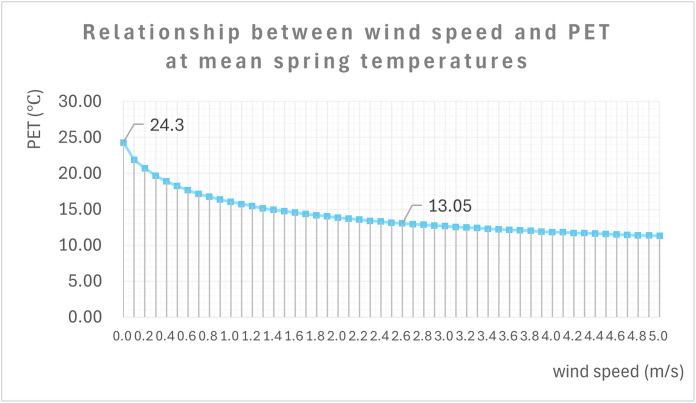
Calculation of human thermal sensation and wind velocity in Rayman.

PET is influenced by meteorological factors such as air temperature, wind velocity, global radiation, and relative humidity, as well as individual factors like age, height, weight, clothing, and activity level. This enables a more comprehensive calculation of the thermal comfort range for outdoor human activities. This paper focuses on the effect of wind velocity variations on outdoor thermal comfort in specific meteorological environments, which can be calculated using Rayman software. The study uses a 35-year-old inhabitant of standard height and weight as the research subject. The relevant parameters are set as shown in Table 5, and the relationship between the inhabitant’s physiological equivalent temperature (PET) and wind velocity changes is shown in Fig 9.

**Table 5:** Input conditions calculated with Rayman.

Air temperature (°C)	Global radiation(w/㎡)	Relative humidity(%)	age	Height(m)	Weight(kg)	clo	Activity(W)
16.01	309.25	42.46	65	1.7	60	1	80

As shown in Fig 9, wind velocity is negatively correlated with PET temperature for a given temperature and humidity. When wind velocity is 0 m/s, the residents’ PET temperature is 24.3°C, and when wind velocity reaches 5 m/s, it drops to 11.7°C. This indicates that PET temperature decreases significantly with wind velocity, with the rate of decrease slowing over time. According to the PET Physiological Equivalent Temperature Evaluation Table [53], when PET is between 13°C and 29°C, the human body experiences thermal comfort [52]. Fig 9 shows that, in spring, the wind velocity range required for residents of this area to maintain thermal comfort is 0–2.6 m/s.

### 2.6. Sensitivity analysis

To ensure the robustness of the design strategy across varying climatic conditions, a sensitivity analysis was conducted.

This study primarily used the meteorological conditions of May (wind velocity: 6.03 m/s; wind direction: 315°) as the representative month for analysis. To further validate the effectiveness of the design under alternative local wind conditions, sensitivity analyses were conducted on wind velocity.

The Table 6 shows the frequency distribution of wind velocities observed in May. As shown in the Table 7, the standard deviation of wind velocity in May (σ = 2.17) was used as the basis for variation to assess the performance of the design under varying wind velocity scenarios. When a fluctuation range of ±2σ was applied, 91.78% of the wind velocity cases for the month were covered.

**Table 6:** Percentage of wind velocity in May.

Wind velocity range (m/s)	0 ~ 1	1 ~ 2	2 ~ 3	3 ~ 4	4 ~ 5	5 ~ 6	6 ~ 7	7 ~ 8	8 ~ 9	9 ~ 10	>10	Sum
**Count**	3	11	15	19	10	10	17	15	2	2	0	104
**Percentage (%)**	22.88	10.58	14.42	18.27	9.62	9.62	16.35	14.42	1.92	1.92	0	100.00

**Table 7:** Percentage of wind conditions in May.

	Formula	Range	Coverage probability
v ± 0.5σ	6.03 ± 1.09 m/s	4.94 ~ 7.12 m/s	32.88%
v ± 1σ	6.03 ± 2.17 m/s	3.86 ~ 8.20 m/s	50.68%
v ± 1.5σ	6.03 ± 4.26 m/s	1.77 ~ 9.29 m/s	72.60%
v ± 2σ	6.03 ± 4.34 m/s	1.69 ~ 10.37 m/s	91.78%

The relative sensitivity of changes in incoming winds to the effects of the program is represented by:


$$S_{a}=\frac{\text{d}S_{c}}{\text{d}V_{\text{0}}}$$

where $\text{d}S_{c}$ is rate of change of wind comfort area (v < 2.6m/s); $\text{d}V_{\text{0}}$ is rate of change of initial incoming wind.

## 3. Result

### 3.1. Feasibility validation

#### 3.1.1. Grid independence validation.

To verify grid independence in the CFD calculations, simulations were conducted at the 1.5 m height wind velocity at the key measurement point P1 using three systematically refined grids. These grids were designated as the fine grid (Grid 1, with 1.39 × 10^6^ cells), medium grid (Grid 2, with 2.09 × 10^6^ cells), and coarse grid (Grid 3, with 3.14 × 10^6^ cells). The grid refinement ratio was set to 1.5, exceeding the official recommendation of 1.3, to ensure that numerical errors could be effectively evaluated. The GCI calculation involved Apparent order of convergence (p) and the final GCI value (GCI_fine_). All simulations were performed under identical conditions for all other parameters. The results are summarized in Table 8.

**Table 8:** Result of grid convergence.

Parameter	Φ1(m/s)	Φ2(m/s)	Φ3(m/s)	r	p	F_s_	GCI_fine_
**Value**	1.33	1.40	1.70	1.5	2.82	1.25	3.07%

As shown in the Table 8, the fine grid yielded a GCI value of 3.07%, which is below the 5% threshold. This indicates that the grid strategy employed in this study has achieved grid convergence, and the numerical simulation results are reliable and independent of grid resolution.

#### 3.1.2. Model validation.

To enable comparison between experimental and simulation results, the model must be constructed based on actual site conditions, including appropriate vegetation and building representations. In this study, Eddy3D was selected as the simulation platform, employing the standard k–ε turbulence model and a logarithmic wind profile as boundary conditions. The simulation used atmospheric boundary layer (ABL) flow as the initial physical environment, with a surface roughness of 0.22. The terrain was refined at 3 levels, totaling 2.09 × 10^6^ cells. The height of the measuring point is 1.5m above the ground level. Setting the inlet wind velocity to be the average of the same period of the actual measurement. 5.5m/s.

**Fig 10:**
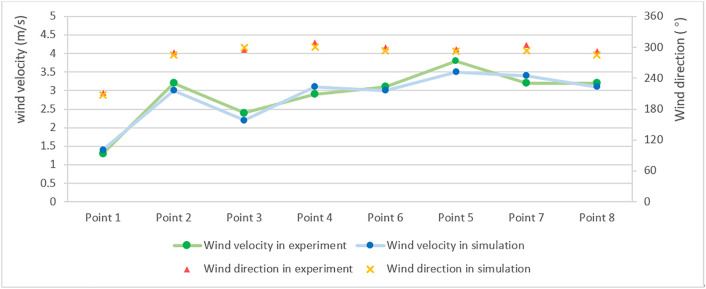
Comparison of wind velocity and direction between experiment and simulation.

Field measurements were integrated with CFD simulation to conduct numerical analysis. The average wind velocity at each measurement point was calculated and compared with experimental data. The results are summarized in Fig 10.

As shown in Table 9, the measured and simulated data exhibited strong agreement. The deviation between simulated and measured wind velocities ranged from 3.13% to 8.33%. The primary sources of error are assumed to be local flow deviations caused by unmodeled features such as eaves and balconies, discrepancies between the vegetation model and actual conditions, inherent instrument error, and transient turbulence effects.

**Table 9:** Comparison of wind velocity between experiment and simulation.

	Point 1	Point 2	Point 3	Point 4	Point 5	Point 7	Point 8
Wind velocity in experiment (m/s)	1.3	3.2	2.4	3.1	3.8	3.2	3.2
Wind velocity in simulation (m/s)	1.4	3.0	3.2	3	3.5	3.4	3.1
Error	7.69%	6.25%	8.33%	3.23%	7.89%	6.25%	3.13%

Based on this quantitative validation, the Eddy 3D simulation effectively captured the spatial characteristics of the wind field within the residential area. It showed notable advantages in predicting wind direction at the pedestrian height of 1.5 meters. The applicability of this simulation approach has also been validated by other researchers [54–56]. Therefore, this method is suitable for subsequent simulations of outdoor wind comfort and safety within residential communities.

### 3.2. Simulation and analysis of the current status of the wind environment at the base

**Fig 11:**
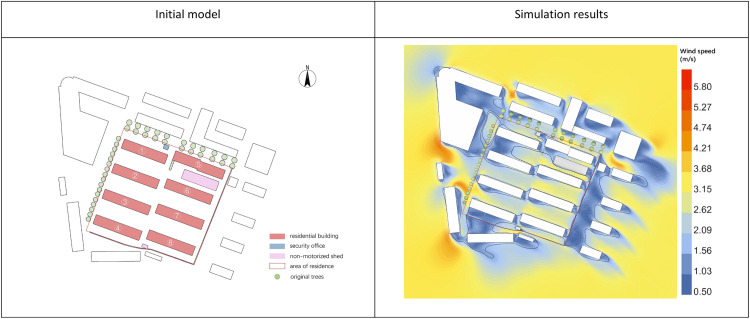
Initial computational modeling and and simulation results.

This study analyzed meteorological data from Zhangjiakou City (2007–2021) and focused on improving the pedestrian-level wind environment in the Tanji Dormitory Area under typical May conditions: NNW315° wind direction at 10 m height and 6.03 m/s wind velocity, according to relevant standards. The simulation used atmospheric boundary layer (ABL) flow as the initial physical environment, with a surface roughness of 0.22. The terrain was refined at 3 levels, totaling 2.09 × 10^6^ cells. The height of the measuring point is 1.5m above the ground level. The RNG k-ε turbulence solver in EDDY 3D was used for computation, with the results shown in Fig 11.

As shown in Fig 11, the primary windward side is the commercial building on the northwest, with incoming wind flowing into the community through the gap between the external buildings. The wind velocity in the simulation area ranges from 0.8 to 4.5 m/s at a pedestrian height of 1.5 m, with higher wind velocities indicated in orange and lower velocities in blue. The building layout in rows and columns creates the Venturi effect, increasing wind velocity between the buildings, with a maximum value of 4.5 m/s, which reduces outdoor comfort. The wind velocity is lower on the short leeward side of the buildings, where residents often rest. In terms of wind direction, the community’s regular layout shields incoming winds with external buildings, directing airflow along the long sides of the buildings.

Table 10 shows that in May, the wind velocity range in the community is between 0.8 and 4.5 m/s, with an average wind velocity of 2.14 m/s, and certain areas exceeding the maximum wind comfort velocity for residents. The community’s wind velocity ratio is 0.54, wind velocity dispersion is 1.022, and the wind comfort zone occupies 40.26% of the area, indicating that the wind velocity in the outdoor space is high and unevenly distributed, resulting in poor wind comfort.

**Table 10:** Analysis of indicators for evaluating the simulation results in the horizontal direction of the initial model of the community.

Wind velocity Max (m/s)	Wind velocity Min(m/s)	Average wind velocity(m/s)	Area of wind comfort(m²)	Average wind velocity ratio	Wind velocity dispersion	Wind comfort area ratio (%)
4.5	0.8	2.14	5548.38	0.539797249	1.022226212	40.26%

### 3.3. Optimization direction and strategy analysis

The aged residential communities are mostly regular rows and columns layout, which under strong wind conditions, result in poorer wind environments compared to open spaces. This is due to the Venturi effect, which accelerates airflow between buildings, negatively impacting outdoor comfort. Two main solutions to this issue are altering the building layout or increasing the spatial roughness of the urban canyon. In the renovation of aged residential communities, building layout has become fixed, making it impractical to increase building spacing. Therefore, the focus shifts to increasing spatial roughness through open spaces, greenery, vertical greening, etc., to buffer the wind effect in narrow spaces [57] This paper examines walls [26], enclosures [58] and vegetation landscaping [59] —key components of outdoor spaces—to assess the impact of their layout on wind conditions and propose practical, easily implementable measures for improving outdoor wind comfort.

#### 3.3.1 Wall modules design.

Erecting a windbreak wall in areas with high outdoor wind velocities can improve the local wind environment. As the wind passes through, a wind shadow area forms behind the wall, significantly altering both wind velocity and direction. To simulate the comfort of the wind environment at pedestrian height, the wall was positioned perpendicular to the prevailing wind direction in this study. The prevailing spring wind velocity is 6.03 m/s. The height of a single windbreak wall is designed to be 2.5 m, or 3 m when enclosed, considering human scale and activity. This design aligns with the outdoor public space structural code (GB51192−2016), which seeks to optimize wind conditions at pedestrian height.

Another commonly used landscape structure is a wall opening, which enhances the structure while promoting air circulation at the wall’s base, preventing pollutant accumulation, and reducing wind pressure to some extent [60]}. The wall opening optimizes the wind environment by creating a gap near the ground that accelerates airflow. This study does not open the entire wall but focuses on a hole located at a height of 2.5 meters above the ground to investigate its effect on the wind environment. Simulation tests reveal that the strength of the vortex formed behind the wall does not affect the lower-body comfort of individuals when a crowd is present. This design takes into account the knee height of the user and the standing body size of Chinese adults (GB/T 10000−2023), ensuring that the gap at the wall’s base remains less than 0.5m.

**Fig 12:**
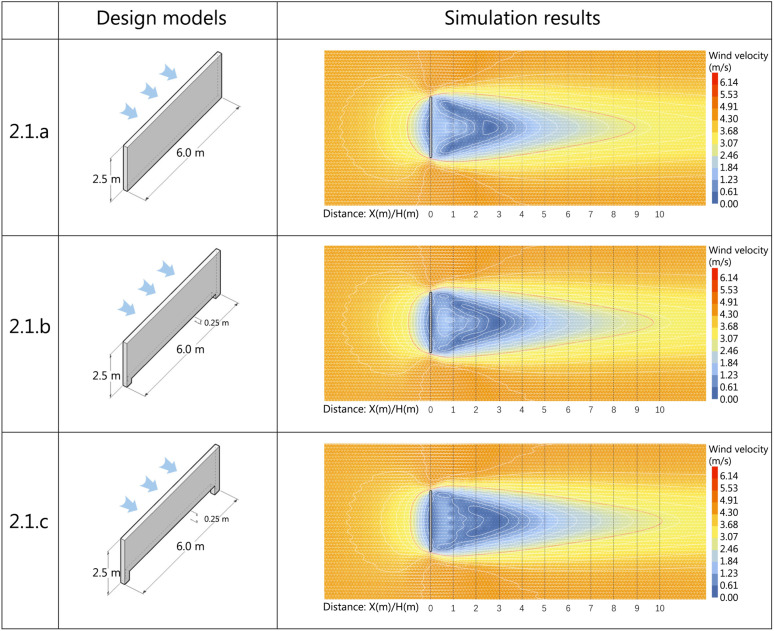
Horizontal wall modules scheme and simulation results.

3.3.1.1 **Horizontal wind condition analysis**. The simulation experiment was conducted with a wind direction of 290° and a wind velocity of 6.03 m/s. The simulation utilized the horizontal plane at a height of 1.5 m as the measurement surface, with the upper limit of wind comfort for residents set at 2.6 m/s at pedestrian height (1.5 m). The wind shadow area is represented by the ratio of the wall height to the leeward distance, denoted as D. The results are shown in Fig 12.

**Fig 13:**
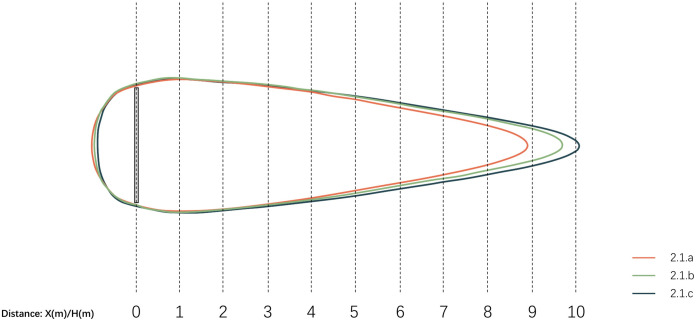
Comparison of horizontal wind comfort zones for wall models.

Fig 13 illustrates that the wind comfort range in Scheme 3.1.a extends to 8.8H, in Scheme 3.1.b to 9.7H, and in Scheme 3.1.c to 10.1H, with the wind comfort study range defined as wind velocity not exceeding 2.6 m/s. This suggests a positive correlation between the wind comfort range at 1.5 m and the height of the open hole, meaning that the higher the open hole at the bottom of the wall, the greater the wind comfort range within the wind shadow area behind the wall.

As shown in Table 11, both the wind comfort area and the static wind area within the wind shadow range increased as the height of the hole increased. Specifically, scheme 3.1.c, with a 0.5-meter high hole at the bottom, achieves the maximum wind comfort area without compromising the comfort of the lower body for individuals behind the wall. The average values of the wind shadow area and wind velocity ratio decreased when the hole at the bottom of the wall was raised to a height of 0.25 meters. This suggests that positioning the hole at the bottom of the wall is an effective method for reducing wind velocity. This may be related to the airflow entering the wind shadow area through the hole, and further research is needed to explore the vertical variation of the wind field in this context.

**Table 11:** Analysis of evaluation indicators for horizontally oriented wall module programs.

	Area of wind comfort(㎡)	Average wind velocity(m/s)	Average wind velocity ratio	Wind velocity dispersion
3.1.a	127.4093	2.70	0.679682949	1.344909100
3.1.b	137.3734	2.43	0.611355033	1.397665692
3.1.c	142.0287	2.49	0.626818720	1.404243569

**Fig 14:**
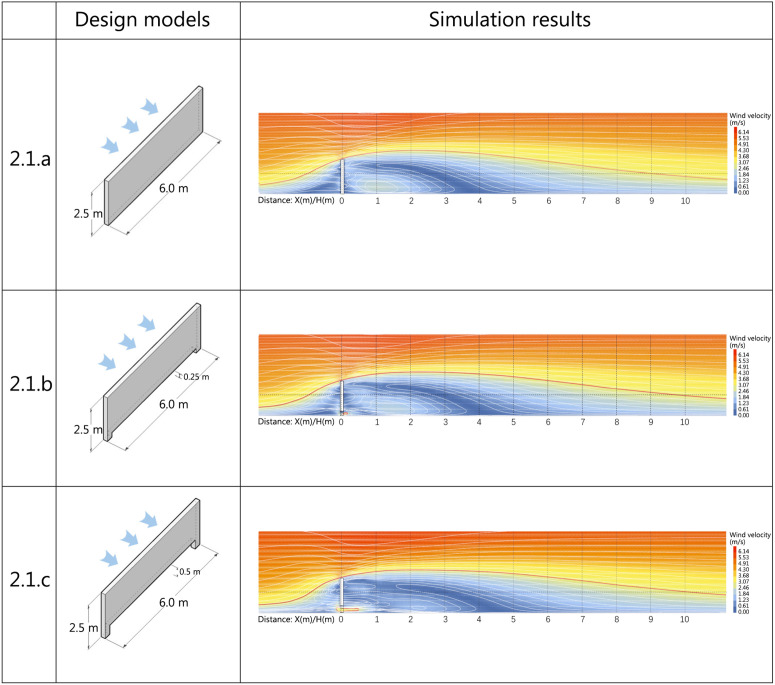
Vertical wall modules scheme and simulation results.

3.3.1.2 **Vertical wind condition analysis**. The horizontal analysis shows little variation in the results. To further assess the impact of aperture height on leeward airflow, a supplementary vertical plane analysis is conducted. Therefore, the vertical plane along the central axis of the windward wall is chosen as the measurement surface, with other parameters kept constant, to investigate how ground-level aperture height variations affect the wind environment. The results are presented in Fig 14.

(1) Analysis of simulation results for Scheme 3.1.a

The airflow is separated into downwash and upwash airflow once the entire wall has obstructed it. Among these, the ground blocked the downwash airflow once more, creating a clockwise downwash vortex that moderated the incoming wind’s flow velocity and created a static wind zone at the wall’s corner of less than 1 m/s; the upwash airflow vortex moderated the incoming wind’s strength on the wall’s windward side; and the wall blockage raised the isopycnic velocity line, as shown in the results that followed. Because of the low pressure, the leeward portion of the wall created a return inverse vortex.

(2) Analysis of simulation results for Scheme 3.1.b

The windward portion of the wall divides the airflow into upwash and downwash, with a hole positioned 20 cm above the ground. The top of the wall generates a high wind velocity area, with a peak value of 4.7 m/s. The downwash and near-ground airflows merge and accelerate into the wind shadow area behind the wall through the hole, where the highest wind velocity is 3.1 m/s. The inflow from the wall hole affects the return vortex in the wind shadow area, tempering the range and size of higher wind velocities. The maximum wind velocity is 2.5 m/s, while the minimum wind velocity (0.7 m/s) remains unaffected at greater distances.

(3) Analysis of simulation results for Scheme 3.1.c

The wall is positioned 50 cm above the ground and contains holes. When the wall blocks the airflow, downwash airflow enters the wind shadow area through the holes. The wind velocity can reach a maximum of 3.4 m/s, with the wind discomfort area extending 0.3 m above the holes and 0.5h behind the wall. Airflow entering through the wall’s holes affects the return vortex in the wind shadow area, with a maximum wind velocity of 1.9 m/s. The minimum wind velocity at a greater distance, 0.7 m/s, remains unaffected.

**Fig 15:**
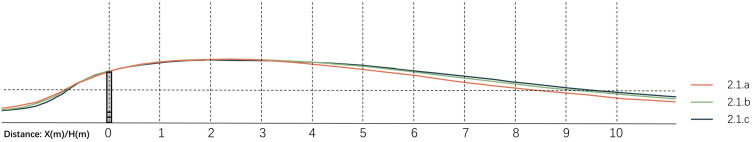
Comparison of Vertical wind comfort zones for wall models.

Fig 15 shows that at 1.5 m elevation, with 2.6 m/s as the comfort threshold, Scheme 3.1.c achieves the longest effective distance (10.1H), while Scheme 3.1.a results in the shortest range (8.8H). This indicates that bottom-wall apertures expand wind comfort zones in leeward regions, with larger aperture heights corresponding to larger comfort areas. This results from the interaction between inflow vortices through apertures and counter-rotating recirculation zones in the wind shadow, reducing wind velocity and expanding comfort zones. Compared to other schemes, Scheme 3.1.c’s 500 mm bottom aperture generates the largest and most uniform wind comfort zone in leeward regions, demonstrating superior wind velocity reduction efficiency. This promotes ground-level airflow circulation, prevents pollutant accumulation, and enhances spatial quality.

#### 3.2. Enclosed spatial modules design.

Outdoor spaces require some enclosed or semi-enclosed areas for leisure and resting. Spaces enclosed by walls offer better wind shielding effects and create larger comfortable wind shadow zones compared to single walls. Based on the actual situation of the Tanji dormitory community, such spaces can also serve as non-motor vehicle parking areas. Therefore, a cubic space with a length and width of 6.6m and a height of 3m was designed to study the impact of different enclosure methods on the wind environment inside and outside the enclosed space.

**Fig 16:**
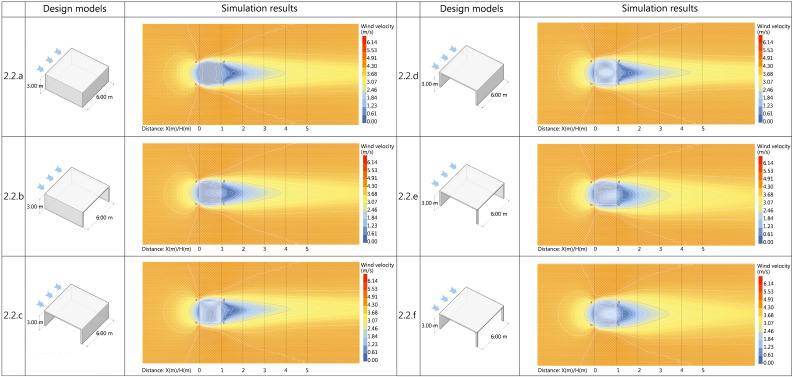
Horizontal enclosed spatial modules scheme and simulation results.

The simulation subject is parallel to the arrangement direction of the buildings in the community. As shown in the figure, six different covered space schemes were designed: fully enclosed, single-sided open with the opening normal direction aligned with the incoming wind, single-sided open with the opening normal direction perpendicular to the incoming wind, double-sided open with adjacent open surfaces, double-sided open with opposite open surfaces, and three-sided open. The wind velocity was set to 6.03 m/s, and the simulation was conducted at a pedestrian height of 1.5m as the measurement plane. The simulation was stopped after convergence was achieved. The range of the wind shadow area is represented by the ratio of enclosed spatial height to leeward distance, denoted as D. The results are shown in Fig 16.

(1) Analysis of simulation results for Scheme 3.2.a

The wall’s directional influence on incoming wind causes airflow concentration at points a and b, resulting in a peak wind velocity of 4.3 m/s. The wind direction shifts away from the main shelter and angles outward, a recurring pattern across all subsequent design iterations. The AB wall’s obstruction generates four low-wind zones (<1 m/s) externally: the AB boundary region where downwash vortices counteract initial airflow, the BC section primarily influenced by low-pressure recirculation vortices, and smaller sections along the CD and DA boundaries. The enclosed spatial form creates complete air stagnation (0 m/s wind velocity), falling within the acceptable range of wind comfort metrics. The trailing airflow in the wind shadow area attains the maximum wind comfort value of 2.6 m/s at 3.95L.

(2) Analysis of simulation results for Scheme 3.2.b

The simulated structure has a unilaterally open design with its aperture normal vector parallel to the wind direction. While maintaining calm wind zones similar to Scheme 1, the low-wind zone along the BC boundary shows reduced coverage. The semi-enclosed configuration with unilateral opening maintains minimal air movement. A pair of counter-rotating recirculation vortices develops symmetrically about the structure’s transverse central axis. The airflow merges at 1.9L on the central axis, realigning with the original wind direction. Velocity escalates proportionally with downstream distance, attaining the geriatrically optimized peak value of 2.6 m/s at 3.75L.

(3) Analysis of simulation results for Scheme 3.2.c

The simulation model adopts a unilaterally open configuration with the aperture normal vector orthogonal to wind direction. This generates a low-wind zone in the semi-enclosed interior, characterized by a counterclockwise rotating vortex that discharges through the CD aperture. The recirculating vortex effectively attenuates corner wind acceleration induced by airflow compression. Concurrently, it reorients the wind shadow zones from BC-edge vortices toward point B, achieving the geriatrically optimized peak wind velocity of 2.6 m/s at 4.15L downstream.

(4) Analysis of simulation results for Scheme 3.2.d

The bilaterally open configuration with opposing aperture orientations generates counter-rotating recirculation vortices — clockwise within the spatial domain and counterclockwise behind the CD wall — symmetrically distributed about the structure’s transverse central axis. The vortex system effectively mitigates corner wind acceleration induced by airflow constriction, consequently expanding wind comfort zones. The geriatrically optimized peak wind velocity of 2.6 m/s occurs at 4.35L downstream, demonstrating enhanced performance in spatial aerodynamics.

(5) Analysis of simulation results for Scheme 3.2.e

The dual-aperture configuration with adjacent openings subjects AB and AD walls to primary wind loading. This generates an asymmetrical pair of recirculation vortices in the semi-enclosed interior, demonstrating distinct flow separation patterns. The low-wind zone reaches maximal spatial coverage at 1.8L, coinciding with airflow re-alignment to the original wind direction. The geriatrically optimized velocity threshold of 2.6 m/s is achieved at 3.45L downstream, validating the aerodynamic efficiency.

(6) Analysis of simulation results for Scheme 3.2.f

The partially enclosed structure, comprising a solitary wall and overhead plane with low enclosure ratio, develops counter-rotating recirculation vortices symmetrically distributed about the transverse central axis within the wind shadow domain. Aerodynamic convergence along the central axis yields localized peak velocity of 1.6 m/s, while downstream progression achieves the geriatrically optimized velocity threshold of 2.6 m/s at 3.4L, demonstrating effective wind regulation.

**Fig 17:**
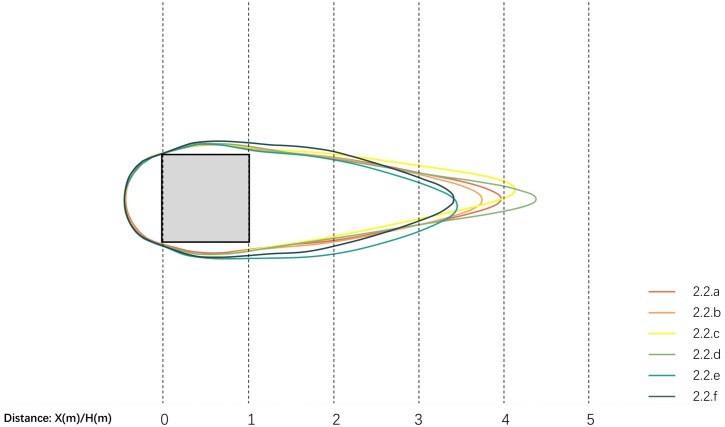
Comparison of horizontal wind comfort zones for enclosed spatial modules.

In terms of wind velocity coverage area, Fig 17 shows Scheme 3.2.d’s open configuration on two opposing sides of the simulated structure generates the largest wind comfort zone. Comparative analysis of enclosure configurations reveals that vertical windbreak stratification and quantity along wind direction predominantly enhance wind comfort zone area. Table 12 shows Scheme 3.2.f has minimal values in average wind velocity, velocity ratio, and dispersion, indicating optimal windbreak performance with uniform wind shadow zone velocity distribution. Analysis of wind shadow zone variations across schemes suggests simpler windbreak configurations yield smaller wind velocity fluctuation amplitudes.

**Table 12:** Analysis of evaluation indicators for horizontally oriented enclosed spatial modules programs.

	Area of wind comfort(㎡)	Average wind velocity(m/s)	Average wind velocity ratio	Wind velocity dispersion
3.2.a	155.2085	2.42	0.609556930	1.452785178
3.2.b	151.3392	2.26	0.569453784	1.498359219
3.2.c	158.2484	2.32	0.584710416	1.424439864
3.2.d	160.8209	2.31	0.582533013	1.374471081
3.2.e	156.9621	2.48	0.624607351	1.368743693
3.2.f	158.3586	2.23	0.561970513	1.401787053

#### 3.3.3. Vegetated landscape modules design.

Evergreen vegetation can reduce wind intensity and create downwind comfort zones, with common windbreak species in Zhangjiakou including juniper, Chinese pine, and arborvitae. The design employs arborvitae with high porosity and compact arrangement as modular units, constructing 2m-diameter models at 2m/4m/6m heights for simulation. The simulation compares wind environment impacts between vertical green systems with varied heights and traditional row-type windbreaks. Three planting schemes were designed: uniform 6m height, ascending (2m-4m-6m) and descending (6m-4m-2m) height gradients. Simulation parameters: 270° wind direction, 4.5m/s velocity, dense porosity, with measurement plane along vegetation axis. The simulation terminated upon achieving convergence criteria.

**Fig 18:**
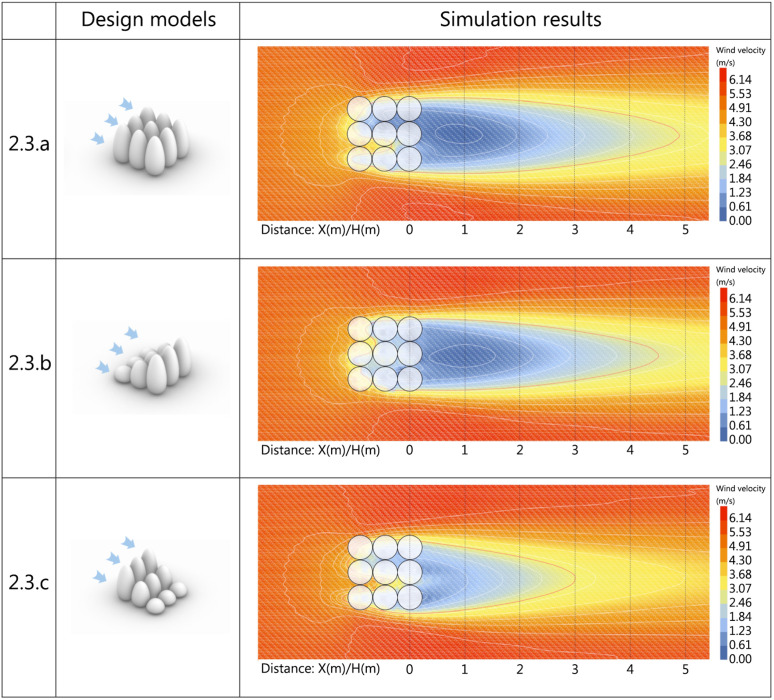
Horizontal vegetated landscape modules scheme and simulation results.

**Fig 19:**
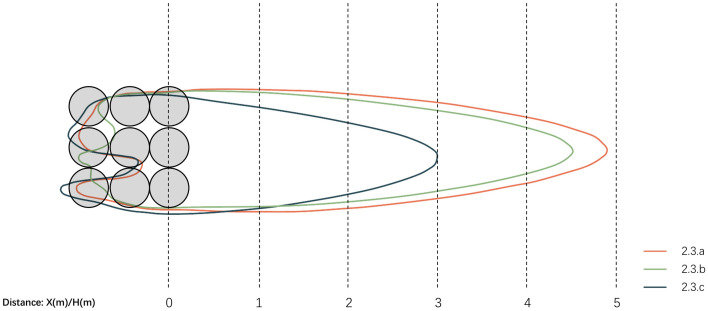
Comparison of horizontal wind comfort zones for vegetated landscape modules.

3.3.3.1. **Horizontal wind condition analysis.** To investigate the variation patterns of horizontal flow fields around windbreak vegetation with different arrangements, simulations were conducted for three configurations: uniform 6m height, ascending (2m-4m-6m), and descending (6m-4m-2m) arrangements. The wind shadow zone range is expressed as the ratio of maximum tree height (6m) to the distance behind the wall, denoted as D. The results are shown in Fig 18. The wind comfort zones for each scheme are integrated and the results are obtained as shown in Fig 19.

Fig 19 shows that with 2.6m/s as the wind comfort threshold, Scheme 3.3.a achieves 4.9D wind comfort range, Scheme 3.3.b reaches 4.5D, and Scheme 3.3.c reaches 3.0D. At 1.5m height, wind comfort range correlates with vegetation arrangement, with uniform height configurations achieving maximum comfort zone extension. Among variable-height arrangements, ascending gradients in wind direction create longer comfort zones than descending patterns.

As shown in Table 13, regarding wind velocity coverage, Scheme 3.3.a’s uniform-height arrangement in the windward direction creates the largest wind comfort zone area in the leeward region. Analysis of mean wind velocity, velocity ratio, and dispersion coefficient shows height-varied configurations yield lower average velocities and smoother velocity gradients in wind shadow zones. To thoroughly investigate spatial wind field characteristics, subsequent analysis will focus on vertical velocity variations across schemes.

**Table 13:** Analysis of evaluation indicators for horizontally oriented vegetated landscape modules programs.

	Area of wind comfort(㎡)	Average wind velocity(m/s)	Average wind velocity ratio	Wind velocity dispersion
3.3.a	223.89041	3.28	0.826043926	1.022553646
3.3.b	194.19154	3.22	0.810944471	0.955569069
3.3.c	140.13617	3.11	0.782054949	1.018146464

**Fig 20:**
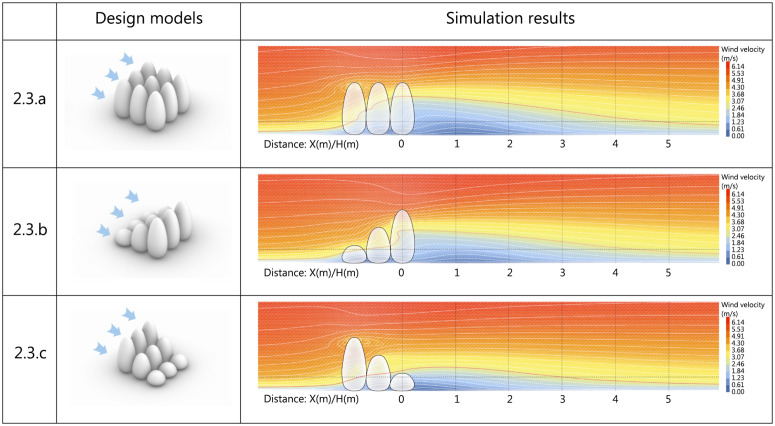
Vertical vegetated landscape modules scheme and simulation results.

3.3.3.2 **Vertical wind condition analysis**. To investigate the vertical flow field patterns around windbreak vegetation with different arrangements, simulations were conducted for three configurations: uniform 6m height, ascending (2m-4m-6m), and descending (6m-4m-2m) arrangements. The results are shown in Fig 20.

(1) Analysis of simulation results for Scheme 3.3.a

The simulated structures are uniformly 6m in height and arranged linearly. At the 1.5m reference plane, wind velocity undergoes significant reduction as airflow passes through the first windward tree. Wind velocity measures 3 m/s at the left edge, 3.2 m/s at the center, and 1.9 m/s at the right edge of the tree. The combined effect of subsequent trees reduces initial outflow velocity to 1.3 m/s, with wind velocity isocontours exhibiting vertical uplift. The wind comfort zone extends to 4.9D behind trees at pedestrian level, with gradual velocity recovery downstream. The porous vegetation model allows airflow penetration without directional deviation, a characteristic consistent across subsequent schemes.

(2) Analysis of simulation results for Scheme 3.3.b

The simulated structures are arranged in a linearly ascending gradient of 2m, 4m, and 6m. As shown in the figure, at the 1.5m reference plane, wind velocity measures 3.3 m/s at the left edge of the first tree, decreasing to 2.9 m/s at the center and 2.7 m/s at the right edge. The limited vertical coverage of the first tree’s canopy at this height reduces horizontal wind shielding effectiveness in the windward zone. Downstream vegetation arrays reduce initial outflow velocity to 1.6 m/s. The wind comfort zone extends 4.5D leeward at pedestrian level, with gradual velocity recovery.

(3) Analysis of simulation results for Scheme 3.3.c

The simulated structures are arranged in a linearly descending gradient of 6m, 4m, and 2m. As shown in the figure, at the 1.5m reference plane, wind velocity measures 2.8 m/s at the left edge of the first tree, decreasing to 3.3 m/s at the center and 1.9 m/s at the right edge. The combined effect of subsequent trees reduces initial outflow velocity to 1.6 m/s. The wind comfort zone extends 3.0D leeward at pedestrian level, with gradual downstream velocity recovery.

Vegetation reduces near-ground wind velocity through mechanisms of aerodynamic resistance and energy dissipation. Branches and leaves act as small obstacles along the airflow path, where inevitable collisions generate considerable surface frictional drag. Moreover, when airflow is forced to circumvent these irregular structures, high-pressure zones develop on the windward side and low-pressure vortex zones on the leeward side, producing strong pressure drag due to the resulting pressure differential. The fundamental effect of these two forms of drag lies in energy conversion: the wind’s kinetic energy is largely transformed into heat and other forms—such as turbulent kinetic energy—through sustained collisions with foliage, friction, and the maintenance of leeward vortices, thereby directly reducing wind velocity [61–63].

As airflow passes through canopy porosity, the originally concentrated and high-velocity stream is dispersed into numerous smaller jets with lower velocities and varying trajectories. The total wind momentum is redistributed across more and finer flow paths, naturally reducing the average velocity within each. In addition, the geometric structure of the porosity redirects portions of the airflow upward or laterally (as observed in the above results, the 2.6 m/s airflow showed upward shifts in isovelocity contours when passing through vegetation). This further reduces the effective penetration velocity of the airflow.

Due to the presence of canopy porosity, airflow does not entirely bypass the crown but partially penetrates through it. During penetration, substantial kinetic energy is lost due to drag and turbulence, resulting in significantly reduced wind velocity on the leeward side. This results in a much smaller pressure gradient between the leeward and windward sides compared to that behind a solid barrier. Moreover, the airflow that penetrates the canopy continuously feeds into the leeward zone, generating a forward-moving stream, which inhibits the formation of reverse recirculating flows, thereby preventing the development of re-circulating vortices induced by low-pressure zones behind the vegetation.

**Fig 21:**
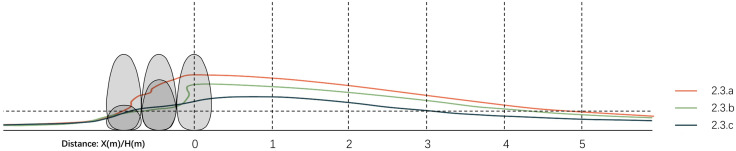
Comparison of vertical wind comfort zones for vegetated landscape modules.

Fig 21 indicates that at 1.5m elevation with 2.6 m/s as the comfort threshold, Scheme 3.3.a achieves the longest effective distance (4.9D), followed by Scheme 3.3.b (4.5D), while Scheme 3.3.c yields the shortest impact range (3.0D). Therefore, when planting shrubs for windbreak purposes, uniform-height and ascending-height arrangements in windward direction are preferable. The former maximizes wind comfort zone coverage, while the latter ensures gentler velocity gradients in leeward zones and enhances spatial diversity.

### 4. Simulation and analysis of modules applications

**Fig 22:**
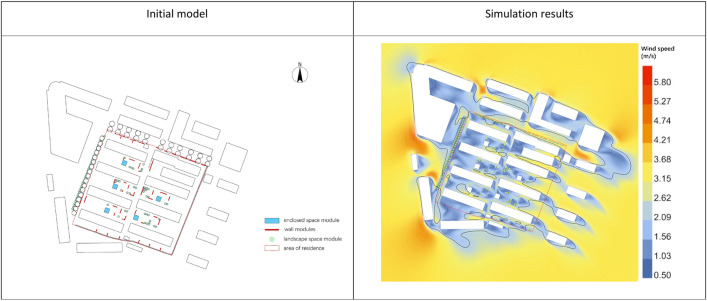
Community transformation program model and simulation results.

Based on the aforementioned optimization methods, corresponding design modules were added to the original model, and the simulation was recalculated under the same conditions. The results are shown in Fig 22.

Based on a comprehensive analysis of the simulation results, it can be observed that the overall wind velocity within the community has significantly decreased. Previous corner winds in multiple locations have been eliminated, and wind velocities in activity areas between buildings and northern entry zones have been reduced overall. The optimized wind environment is more suitable for outdoor activities.

Table 14 shows the wind velocity within the residential area ranged from 0.8 to 3.6 m/s, with an average of 1.46 m/s. In most areas, wind velocities were below the upper threshold of wind comfort for elderly individuals. The wind velocity ratio was 0.369, and the velocity dispersion was 0.446. The wind comfort area accounted for 79.84% of the total outdoor space. These results indicate that under the prevailing spring wind velocity of 6.03 m/s, the overall wind velocity in the outdoor space was effectively reduced, with a more uniform distribution, indicating an improved wind environment.

**Table 14:** Analysis of indicators for evaluating the wind environment in a modified communityTo assess the effectiveness of the design under varying wind velocities in the study area, the standard deviation of the prevailing wind velocity in May was calculated as σ = 3.17. Comparative analyses of the design performance were conducted under wind velocity fluctuations of ±0.5σ, ± 1σ, ± 1.5σ, and ±2σ, respectively. The results are shown inTable 15.

Wind velocity Max (m/s)	Wind velocity Min(m/s)	Average wind velocity(m/s)	Area of wind comfort(m²)	Average wind velocity ratio	Wind velocity dispersion	Wind comfort area ratio
3.6	0.8	1.464516129	11003.06	0.368669126	0.446013063	79.84%

As shown in the Table 15, changes in incoming wind velocity led to corresponding variations in the proportion of wind-comfortable areas. When wind velocity gradually decreased from the baseline of 6.03 m/s in 0.5σ intervals, both the pre- and post-intervention wind comfort areas increased. At a reduced wind velocity of 2.77 m/s, the pre-intervention comfort area reached full coverage, and the proposed design did not have a negative impact. Conversely, as wind velocity increased by 0.5σ intervals above 6.03 m/s, the proportion of comfortable areas decreased under both scenarios. However, in terms of the change in wind comfort area proportion, the design consistently improved wind comfort regardless of whether wind velocity increased or decreased, demonstrating its broad applicability. Sensitivity coefficient analysis further revealed that when wind velocity decreased incrementally (by 0.5σ), the absolute values of the sensitivity coefficients were less than 1, indicating that wind velocities below 6.03 m/s were non-sensitive factors for wind comfort improvement. In contrast, when wind velocity increased by 0.5σ intervals, the coefficients exceeded 1 in absolute value, suggesting that wind velocities above 6.03 m/s were sensitive factors affecting the scheme’s effectiveness. Although stronger winds in this range weakened the enhancement effect, Table 6 shows they accounted for only 34.62% of the prevailing wind frequency and thus did not significantly impact the overall conclusions under typical spring conditions.

**Table 15:** Analysis of indicators for evaluating the wind environment in a modified community.

Incoming wind velocity	1.69	2.77	3.86	4.94	6.03	7.12	8.2	9.29	10.37
**formula**	6.03−2σ	6.03-1.5σ	6.03−1σ	6.03-0.5σ		6.03 + 0.5σ	6.03 + 1σ	6.03 + 1.5σ	6.03 + 2σ
**Percentage of wind comfort area before remodeling**	100.00%	100.00%	99.63%	56.07%	40.19%	18.13%	9.80%	5.40%	1.41%
**Percentage of wind comfort area after remodeling**	100.00%	100.00%	100.00%	93.62%	79.70%	45.41%	20.00%	14.84%	3.52%
**Wind comfort area ratio variation**	25.46%	25.46%	25.46%	17.46%		−43.02%	−74.91%	−81.38%	−95.58%
**Wind velocity ratio variation**	−71.97%	−54.06%	−35.99%	−18.08%		18.08%	35.99%	54.06%	71.97%
**Sensitivity factor**	−0.35	−0.47	−0.71	−0.97		−2.38	−2.08	−1.51	−1.33

## 4. Discussion

### 4.1. Design rationale and strategic intervention

This study aims to design a prototype for the layout of outdoor facilities to mitigate wind velocity in the outdoor environments of aged residential communities during strong wind events, thus enhancing the thermal comfort of individuals engaged in outdoor activities. Furthermore, the final simulation results indicate a notable improvement, with the design seamlessly integrating into the environment and fostering a positive spatial experience, offering valuable insights for designers working on environmental engineering in areas prone to strong winds.

Most studies on human comfort in outdoor environments concentrate on the integrated assessment of elements within relevant thermal indicators. However, a study by Y et al. revealed that in cold regions, air temperature, solar radiation, wind, and humidity contribute 56%, 29.4%, 8.8%, and 5.9%, respectively, to outdoor thermal sensation [64]. In regions such as Zhangjiakou City, which experience frequent strong winds year-round, wind velocity significantly affects human comfort. China has established standards for wind velocity in outdoor environments, but these are confined to universal designs for general climates and provide little guidance for specific regions with extreme weather conditions. This study, targeting the unique climate of Zhangjiakou City, calculated a comfort model for residents in outdoor environments. Based on Höppe’s research, which identified a thermal neutral temperature range of 13–29°C [53], the suitable wind velocity for outdoor environments was derived as no more than 2.6 m/s, meeting the local residents’ demand for a healthy community wind environment.

The study reveals that high-wind-velocity zones in outdoor environments of aged residential communities primarily stem from two factors: first, the deflection of wind by external buildings leads to elevated wind velocities at the primary windward facades; second, the Venturi effect in narrow internal spaces generates accelerated airflow.Mitigation strategies for the first factor involve deploying wind barriers—such as walls, baffles, or multi-layered vegetative screens—at windward edges. It should be noted that due to recirculating vortices formed in low-pressure zones of the wind field, the effectiveness of these windbreaks is limited to specific ranges. Beyond these zones, wind velocities gradually recover to initial velocities. Consequently, spatial arrangement of windbreaks requires rigorous analysis of their effective radii. Three prototype systems—wall units, enclosed spatial modules, and vegetated landscape elements—were designed and evaluated for their impact ranges, offering actionable guidelines for landscape architects. Addressing the second challenge requires regulating distances between structures and installations to prevent tight clearances that amplify wind velocities through tunnel effects.

### 4.2. Sustainability and maintenance considerations

In addition, the proposed wind-mitigation infrastructure must also confront the challenges of long-term operation and maintenance.

The sustainability of windbreak wall relies on material innovation and structural design. Frequent strong winds and salt-alkali erosion in the Zhangjiakou region easily lead to surface cracks and structural damage of the wall. To address this, polymer-filled panels can be added to the concrete structure to enhance strength and rigidity [65]. More crucially, modular design involves decomposing the wall into small, replaceable units, so that only the damaged module needs to be replaced in case of local damage, which reduces overall maintenance costs and minimizes construction waste.

In vegetation modules, maintenance costs and ecological adaptability are key limiting factors. Based on ecological restoration practices in cold regions, it is recommended to prioritize deep-rooted native shrubs as the main species, as their drought resistance reduces irrigation dependence and, through related management mechanisms, controls the replanting rate. Although the vegetation composition requires medium-term maintenance, its initial investment is lower than that of concrete barriers and provides additional carbon sequestration benefits [66]. This “ecology-based engineering” model provides a new pathway for low-cost wind environment renovation in aged residential communities

### 4.3. Socio-Cultural dimensions and design equity

This study significantly improved the wind environment comfort in aged residential communities through multi-module collaborative design, but it is important to acknowledge the limitations of the current research: comfort interventions are not only an optimization of physical parameters but also a practical vehicle for social equity.

In terms of environmental equity, the layout of wind barriers must be cautious of resource allocation imbalances. Fixed facilities occupy limited public space, leading to reduced activity areas, fewer parking spaces, or broken pedestrian pathways. In space-constrained aged residential communities, wind protection needs easily conflict with functions such as fitness and recreation; at the same time, the wind comfort needs of different age groups (such as the elderly and children) have not been adequately considered in the design. The elderly sitting area and the children’s activity area have different sensitivities to wind velocity, and a unified design may lead to insufficient optimization of the experience for certain groups. Therefore, achieving spatial justice for wind barriers requires, as a primary premise, breaking the monopoly of technical decision-making, which requires in-depth field interviews to deeply engage residents in community planning: based on the residents’ wind environment pain point map (with a focus on the needs of the elderly, children, and low-income groups), priority should be given to ensuring the wind protection needs of vulnerable groups in spatial resource allocation, for example, placing small-scale, multifunctional wind barrier units (such as ventilated grille walls combined with resting seats) in frequently used residential open spaces, rather than mechanically adhering to the optimal efficiency principles of wind engineering. Design standards should establish a differentiated wind comfort grading system—with wind velocities in sitting areas for conversation to be below 2.6 m/s, in children’s activity areas to be relaxed to above 2.6 m/s, and pedestrian pathways to retain moderate airflow for air renewal, and dynamically responding to the contrasting wind environment needs of winter and summer through flexible facilities such as rotating panels and seasonal vegetation.

In the social cognitive dimension, the acceptance of facilities is highly dependent on cultural adaptability. Although this study prioritizes Juniperus chinensis as the core species for vegetation modules based on ecological efficiency, its singular form and seasonal characteristics can easily lead to aesthetic fatigue or even spatial avoidance behavior among residents, reflecting a disconnect between scientific rationality and community cultural identity. To bridge this gap, future designs must urgently reconstruct the decision-making process through participatory community planning—inviting residents to jointly select native plants that combine wind protection performance with cultural affinity, and design a multi-seasonal landscape sequence, to achieve a dynamic balance between ecological function and regional aesthetics. At the same time, visual interventions in wind barriers require particular caution: when the enclosing height is relatively high, its physical barrier property can easily be transformed into a symbol of psychological oppression, especially creating negative perceptions for lower-floor residents and in confined spaces [67]. In response, artistic translation strategies can be used to alleviate the confrontational effect: incorporating local traditional patterns and paintings on the surface of concrete prefabricated components, using gradient-colored paint to soften the sense of mass, or using perforated screens to achieve both wind direction flow and visual permeability goals. These measures not only reduce the environmental intrusion of the facilities but also turn wind barrier projects into cultural carriers that embody collective memory, ultimately achieving synergistic benefits between technical efficacy and social recognition.

### 4.4. Prospects for subjective perception research

The above discussion highlights a potential gap between technological effectiveness and social acceptance in environmental interventions. To systematically bridge this divide and address the crucial dimension of subjective perception, we propose a comprehensive pilot study. This study aims to integrate the optimization of the built environment’s physical performance with residents’ socio-psychological experiences. Beyond supplementing and validating the effectiveness of physical interventions, the plan places social equity and resident well-being at the core of the evaluation framework, ensuring that technological solutions genuinely serve human needs. Through this initiative, we aspire to establish an assessment paradigm that combines scientific rigor with humanistic care, providing valuable guidance for future community renewal projects.

The first phase of this plan focuses on establishing a comprehensive baseline database. Data will be systematically collected through a combination of structured questionnaires and semi-structured in-depth interviews. The questionnaires will employ standardized Likert scales to quantitatively assess residents’ satisfaction with the current outdoor wind environment, frequency of space usage, sense of safety, and overall comfort. In parallel, the in-depth interviews will target different demographic groups—particularly the elderly and children—to map their specific “pain points” and gain a deeper understanding of how the wind environment affects their daily activities and emotional connections.

In the early period following the interventions, the second-phase assessment will be initiated promptly to capture residents’ initial responses and adaptation. Within 1–3 months post-intervention, the questionnaires and follow-up interviews will be repeated, with a focus on evaluating residents’ aesthetic acceptance of newly constructed elements (e.g., windbreak walls and enclosed spaces), their functional utility, and the perceived improvements in the wind environment. To enhance objectivity, this phase will also incorporate on-site behavioral observations. By recording actual usage patterns—such as duration of stay and frequency of social interactions—these observations will provide a cross-validation of the interview and questionnaire findings.

To assess the long-term sustainability and social impact of the interventions, the third-phase evaluation will be conducted across different seasons following project completion. This phase shifts the focus from short-term adaptation to long-term integration, examining aspects such as the maintenance status of the facilities, residents’ care and stewardship, and whether the newly created spaces have successfully evolved into active community social nodes. Importantly, we will also analyze residents’ seasonal variations in needs—such as during hot summers and cold winters—to comprehensively evaluate the design’s all-weather adaptability and its overall value throughout the lifecycle.

## 5. Conclusion

This study investigates the wind comfort of outdoor spaces in residential areas in regions with frequent strong winds. Innovatively differing from quantitative comfort calculations in other microclimate environments, it proposes a series of windbreak facility prototypes to improve outdoor wind comfort. CFD simulations were conducted for 12 design schemes under three outdoor element types: walls, enclosed spaces, and vegetation landscape spaces. Most wind velocity zones were maintained below 2.6m/s, and the reduction in pedestrian-level wind velocities increased residents’ equivalent physiological temperature, thereby improving perceived comfort. The results indicate that:

1. Creating openings at the bottom of outdoor windbreak walls enhances airflow in stagnant wind areas at the corners, reducing pollutant buildup. Moreover, increasing the height of the openings at the wall’s base extends the reach of the wind shadow zone, effectively expanding the wind comfort area.
2. For cubic outdoor enclosed spaces, the enclosure method influences the wind field differently. The key factors improving wind comfort area are the number and arrangement of windbreaks perpendicular to the wind direction. The fluctuation of wind velocity in the wind shadow zone depends on the windbreak’s shape, with simpler forms leading to less variation in wind velocity.
3. For windbreak considerations in vegetation landscape design, evergreen plants with high porosity, like juniper, are chosen. Densely arranged plants achieve optimal windbreak efficiency when aligned at equal heights in the windward direction. A height-increasing arrangement in the windward direction, though less effective, lowers economic investment and enhances spatial comfort.
4. After applying the three strategies mentioned above to improve the outdoor space of aged residential communitie**s**, simulated by computer, under prevailing wind velocities in spring, the outdoor wind comfort area increased from 40.26% to 79.84%, significantly improving the wind comfort in the residential outdoor areas.

This study, through the CFD simulation-based wind environment optimization design prototype for residential communities, demonstrates significant improvements in wind comfort in areas with strong winds. Future research could supplement with an analysis based on measured data and surveys of different population groups, as well as conducting field measurements and computer simulations in different seasons for comparative experiments, to systematically evaluate the adaptability performance of the design scheme. Methodologically, the applicability of this design prototype in different climate zones and urban spatial forms could be explored. Future research could focus on multi-species vegetation configuration, innovative spatial enclosure, and other synergistic microclimate regulation technologies, developing wind comfort enhancement systems that adapt to diverse built environments. In practical terms, the research outcomes could be introduced into urban planning and residential community renovation through government-university collaboration, using a wind comfort-based evaluation system, incorporating the synergistic optimization of wind protection facilities and landscape design into residential construction technical guidelines, to promote the adoption of wind environment optimization strategies in new and renovation projects, thereby enhancing the livability of outdoor spaces.
