# Correction: Research on wind comfort regulation strategies for public spaces in aged residential communities of cold regions during windy spring seasons

**DOI:** 10.1371/journal.pone.0342587

**Published:** 2026-02-10

**Authors:** Wen Xue, Xiaojun Guo, Xiaodan Chen, Yuanfeng Wang, Rui Chai, Yichao Wang, Yu Xia, Qianjun Cai

There are errors in the Funding statement. The correct Funding statement is as follows: This study was funded by grants from the Department of Education of Hebei Province (QN2023214), awarded to Wen Xue, and the Hebei University of Architecture Innovation Fund (XY2024087).
